# Cortical Thickness and Appetite Hormones in Adolescent Obesity and Binge Eating Disorder: A Comparative Study

**DOI:** 10.1002/eat.70161

**Published:** 2026-06-28

**Authors:** Serkan Turan, İbrahim Mert Erbaş, Fatma Ceren Sarıoğlu, Berrin Çavuşoğlu, Ezgi Molla, Ali Rıza Şişman, Sevay Alsen Güney, Handan Güleryüz, Ayhan Abacı, Yeşim Öztürk, Aynur Pekcanlar Akay

**Affiliations:** ^1^ Department of Child and Adolescent Psychiatry Uludag University School of Medicine Bursa Turkey; ^2^ Department of Pediatric Endocrinology, İzmir Faculty of Medicine Dr. Behçet Uz Children's Hospital İzmir Türkiye; ^3^ Department of Radiology Dokuz Eylul University School of Medicine İzmir Turkey; ^4^ Ataturk Vocational School of Health Services Afyonkarahisar Health Sciences University Afyon Turkiye; ^5^ Department of Biochemistry Dokuz Eylul University School of Medicine İzmir Turkey; ^6^ Department of Child and Adolescent Psychiatry Dokuz Eylul University School of Medicine İzmir Turkey; ^7^ Department of Pediatric Endocrinology Dokuz Eylul University School of Medicine İzmir Turkey; ^8^ Department of Child Gastroenterology Dokuz Eylul University School of Medicine İzmir Turkey

**Keywords:** adolescent obesity, binge eating disorder, brain structure, cortical thickness, ghrelin, MRI, neuropeptide Y

## Abstract

**Objective:**

This study examined cortical thickness and appetite‐regulating hormones—neuropeptide Y (NPY) and ghrelin—to better understand the neurobiological mechanisms underlying binge eating disorder (BED) and obesity in adolescence. We compared adolescents with BED and obesity, adolescents with obesity without BED, and healthy controls (HCs), and explored the relationships among these measures, BMI, and psychological symptoms.

**Method:**

Seventy adolescents (aged 12–18 years) were included: 24 adolescents with BED and obesity, 24 adolescents with obesity without BED, and 22 HC. Cortical thickness was measured using 3.0 T structural MRI with FreeSurfer‐based analysis, and fasting serum levels of NPY and ghrelin were quantified by ELISA. Participants also completed the Eating Attitudes Test‐40 (EAT‐40), the Eating Disorder Examination Questionnaire (EDE‐Q), the Children's Depression Inventory (CDI), and the State–Trait Anxiety Inventory for Children (STAI‐C).

**Results:**

Both groups with obesity showed significantly elevated NPY levels compared to HCs (*p* < 0.001), with no difference between adolescents with and without BED; fasting total ghrelin levels did not differ across groups. Adolescents with BED exhibited reduced cortical thickness in the right posterior cingulate cortex relative to both comparison groups (*p* = 0.002), while left posterior cingulate thinning was observed in both groups with obesity versus HCs (*p* = 0.004). No significant group differences were found in insular or anterior cingulate thickness. Both groups with obesity showed greater depressive symptoms, disordered eating attitudes, and eating‐related psychopathology than HCs. In multiple regression analyses adjusted for diagnostic group membership, higher BMI (*β* = 0.47, *p* < 0.001) and lower left insular cortical thickness (*β* = −0.45, *p* = 0.031) were independently associated with elevated NPY levels.

**Conclusion:**

Findings suggest that elevated NPY levels may represent a shared neurobiological feature of adolescent obesity, whereas reduced right posterior cingulate cortical thickness may be more specifically associated with the diagnosis of BED. These findings contribute to understanding the neurobiological correlates of disordered eating behaviors during adolescence.

## Summary

- This study examined cortical thickness and appetite‐related hormones (neuropeptide Y and ghrelin) in adolescents with obesity with and without binge eating disorder (BED), compared with healthy controls.
- Both groups with obesity showed significantly elevated neuropeptide Y levels relative to healthy controls, whereas ghrelin levels did not differ between groups.
- Adolescents with BED demonstrated reduced right posterior cingulate cortical thickness, while left posterior cingulate thinning was observed in both obesity groups.
- Higher body mass index and lower left insular cortical thickness were independently associated with elevated neuropeptide Y levels after adjustment for diagnostic group membership.
- Findings suggest that elevated neuropeptide Y may represent a shared neurobiological feature of adolescent obesity, whereas posterior cingulate cortical alterations may be associated with the diagnosis of BED.

## 1 Introduction

Binge eating disorder (BED) has emerged as the most prevalent eating disorder in adolescence and represents a clinically significant condition characterized by recurrent episodes of excessive food intake accompanied by a subjective loss of control (American Psychiatric Association 2013). Beyond its behavioral definition, BED reflects a complex interplay of dysregulated appetite signaling, impaired cognitive control, and altered reward processing. Unlike bulimia nervosa, binge episodes in BED are not followed by compensatory behaviors, which contributes to its strong association with weight gain and the development or maintenance of obesity.

Adolescence constitutes a particularly sensitive developmental window for the emergence of BED, given the ongoing maturation of neural circuits involved in impulse control, reward sensitivity, and emotional regulation. During this period, the convergence of biological vulnerability and environmental influences may facilitate the transition from normative variations in eating behavior to clinically significant pathology. Epidemiological data indicate that BED affects approximately 10%–30% of adolescents with obesity, and its presence is consistently associated with greater psychiatric burden, including elevated depressive and anxiety symptoms, as well as poorer metabolic and treatment outcomes compared to obesity alone (McCuen‐Wurst et al. 2018; Reas et al. 2025). These observations suggest that BED in the context of obesity may represent a distinct neurobiological phenotype rather than merely a severity marker of excess weight.

From a mechanistic perspective, growing evidence implicates appetite‐regulating hormones as key modulators of both homeostatic and hedonic feeding processes. Ghrelin, primarily secreted by the stomach, exerts potent orexigenic effects while also enhancing reward‐related responses to food via mesolimbic dopaminergic pathways (Polishchuk et al. 2025). Prior findings regarding ghrelin in BED have been mixed, with several studies reporting lower fasting ghrelin levels in individuals with BED and obesity compared to obesity alone despite positive associations between ghrelin and binge‐eating frequency (Atalayer et al. 2013). Neuropeptide Y (NPY), one of the most powerful hypothalamic peptides regulating energy balance, plays a central role in promoting food intake and modulating stress‐related feeding behavior (Loh et al. 2015). In contrast to ghrelin, human research directly examining NPY differences between BED and obesity without BED remains limited, and its specific role in binge‐eating pathophysiology is not yet well established. Dysregulation of these hormonal systems has been observed in both obesity and eating disorders, supporting the notion that peripheral metabolic signals may interact with central neural circuits to sustain maladaptive eating patterns (Turan et al. 2021). However, whether such alterations specifically differentiate BED from obesity without binge‐eating remains insufficiently understood. Among appetite‐related hormones, ghrelin and NPY were selected because both play central roles in homeostatic and hedonic feeding processes and have been implicated in obesity, reward sensitivity, and dysregulated eating behaviors (Loh et al. 2015; Turan et al. 2021). Ghrelin influences food intake not only through hypothalamic mechanisms but also through mesolimbic reward pathways, whereas NPY is one of the most potent orexigenic neuropeptides involved in appetite regulation and stress‐related eating (Loh et al. 2015; Turan et al. 2021). Despite their biological relevance, relatively few studies have examined these hormones in relation to structural brain measures in adolescents with obesity and BED (Turan et al. 2021).

Converging evidence from structural neuroimaging further highlights the involvement of distributed cortical networks in the regulation of eating behavior. Cortical thickness, a neuroanatomical marker reflecting synaptic organization and cortical maturation, has been shown to be altered in individuals with obesity and eating disorders (Turan et al. 2021). Cortical thickness was prioritized because it is considered a sensitive marker of cortical maturation, synaptic remodeling, and neurodevelopmental change during adolescence (Lotter et al. 2024). Unlike subcortical volumetric measures, which tend to remain relatively stable across this developmental period, cortical thickness undergoes substantial age‐related remodeling and has been linked to behavioral regulation, reward processing, and interoceptive functions relevant to eating behavior (Lotter et al. 2024). Therefore, cortical thickness was considered particularly well suited for investigating neurodevelopmental alterations associated with obesity and BED during adolescence. However, findings regarding cortical thickness alterations in obesity and BED have been heterogeneous, with some studies reporting reduced prefrontal thickness and others suggesting altered developmental trajectories of cortical maturation associated with higher BMI (Turan et al. 2021). Reductions in cortical thickness have been particularly reported in regions implicated in executive control (prefrontal cortex), interoceptive awareness (insula), and salience and conflict monitoring (anterior cingulate cortex) (Friedman and Robbins 2022; Tang et al. 2025). These regions collectively support the integration of internal physiological states with goal‐directed behavior, and their dysfunction may contribute to impaired inhibitory control and heightened responsivity to food‐related cues. In adolescents with BED, such alterations may be especially pronounced, reflecting a disruption in the balance between top‐down regulatory control and bottom‐up reward‐driven processes (de la Cruz et al. 2021).

Despite these advances, a critical gap in the literature lies in the limited integration of neuroendocrine and neurostructural perspectives, particularly in adolescent populations. Most prior studies have examined hormonal or neuroimaging correlates in isolation, often in adult samples, thereby limiting understanding of how peripheral metabolic signals may relate to central neural architecture during a key developmental period (Murray et al. 2022). Given that adolescence is characterized by rapid neurobiological remodeling and increased sensitivity to reward, the interaction between appetite‐related hormones and cortical structure may be especially relevant for understanding vulnerability to BED (Lowe et al. 2020).

Addressing this gap, the present study adopts an integrative neurobiological framework to examine both peripheral (ghrelin and NPY) and central (cortical thickness) correlates of disordered eating in adolescents. Specifically, we compared adolescents with obesity and BED, adolescents with obesity without BED, and healthy controls to disentangle obesity‐related from BED‐specific alterations. We hypothesized that (i) appetite‐regulating hormone levels would be elevated in obesity irrespective of BED status, reflecting a general dysregulation of energy homeostasis, and (ii) adolescents with BED would exhibit additional, region‐specific cortical alterations in brain networks implicated in self‐referential processing and behavioral regulation. Furthermore, we explored whether cortical thickness measures would independently predict NPY levels, providing exploratory insight into potential associations between central neural systems and peripheral metabolic signals during adolescence. By integrating neuroendocrine and neuroimaging approaches within a developmental framework, this study aims to advance current understanding of the neurobiological mechanisms underlying BED in adolescence. Elucidating these mechanisms may have important implications for identifying biologically informed subtypes and developing targeted interventions that address both metabolic and neurocognitive dimensions of disordered eating.

## 2 Method

### 2.1 Participants

Seventy adolescents aged 12–18 years were included in this case–control study. Participants were allocated to three groups: adolescents with obesity and binge eating disorder (BED; *n* = 24), adolescents with obesity without BED (*n* = 24), and healthy controls (HC; *n* = 22). Clinical participants were recruited from outpatient child and adolescent psychiatry services, while healthy controls were recruited from the community.

Obesity was defined as a body mass index (BMI) ≥ 95th percentile for age and sex, according to the Centers for Disease Control and Prevention (CDC) growth charts. BED diagnosis was established by trained child and adolescent psychiatrists using a structured clinical interview based on DSM‐5 criteria. Adolescents in the obesity comparison group were required not to meet current or past DSM‐5 criteria for BED, and individuals with subthreshold or clinically significant loss‐of‐control eating symptoms suggestive of BED were excluded. Diagnostic procedures incorporated information from both adolescents and their parents to ensure clinical accuracy. Healthy controls were required to have a BMI between the 25th and 75th percentiles and no history of psychiatric or chronic medical illness.

Exclusion criteria for all participants included: lifetime diagnosis of psychotic disorders, bipolar disorder, anorexia nervosa, or bulimia nervosa; intellectual disability; neurological disorders; history of traumatic brain injury with loss of consciousness; and current use of medications affecting appetite, weight, or central nervous system functioning (e.g., antipsychotics, corticosteroids). Participants with contraindications to MRI (e.g., metallic implants, claustrophobia) were also excluded.

All participants underwent a comprehensive clinical evaluation prior to inclusion. Written informed consent was obtained from parents or legal guardians, and assent was obtained from adolescents. Study procedures were completed during the clinical evaluation phase. Psychiatric diagnostic interviews and psychometric assessments were administered by trained clinicians in an outpatient setting. Following an overnight fast, venous blood samples were collected between 8:00 and 10:00 a.m. Structural MRI acquisition was performed on the same day whenever feasible or within a short interval after clinical assessments, depending on participant availability and scanner scheduling. Participants were recruited and study data were collected between 2018 and 2023. The study protocol was approved by the local ethics committee and conducted in accordance with the Declaration of Helsinki.

### 2.2 Clinical and Psychometric Assessments

All participants underwent a comprehensive clinical and psychometric evaluation using standardized, validated instruments to assess eating‐related pathology, depressive symptoms, and anxiety. Assessments were administered in a controlled clinical setting under the supervision of trained researchers to ensure consistency and data quality.

Eating attitudes and disordered eating behaviors were assessed using the Eating Attitudes Test (EAT‐40), a widely used self‐report instrument designed to evaluate maladaptive eating patterns, including dieting behaviors, bulimic tendencies, and preoccupation with food. The validated Turkish version of the EAT‐40 was used in this study (Altuğ et al. 2000).

Eating disorder–specific psychopathology was further evaluated using the Eating Disorder Examination Questionnaire (EDE‐Q), which provides a multidimensional assessment across four domains: restraint eating, eating concern, weight concern, and shape concern. The validated Turkish adaptation of the EDE‐Q was administered. The Turkish psychometric evaluations of the EDE‐Q were conducted by Yucel et al. (2011).

Depressive symptoms were assessed using the Children's Depression Inventory (CDI), a widely used self‐report instrument evaluating cognitive, affective, and behavioral dimensions of depression in youth. The Turkish version of the CDI has demonstrated strong reliability and validity, as reported by Öy (1991).

Anxiety symptoms were measured using the State–Trait Anxiety Inventory for Children (STAI‐C), which distinguishes between state (situational) and trait (dispositional) anxiety. The validated Turkish version of the STAI‐C was used in this study. Previous research has reported Cronbach's *α* values ranging from approximately 0.82 to 0.87 for both state and trait anxiety subscales (Özusta 1995).

Psychiatric diagnoses, including the assessment of BED and exclusion of other psychiatric conditions, were established using the Schedule for Affective Disorders and Schizophrenia for School‐Age Children—Present and Lifetime Version (K‐SADS‐PL), a semi‐structured diagnostic interview administered by trained child and adolescent psychiatrists. The Turkish version of the K‐SADS‐PL has demonstrated good inter‐rater reliability and validity in clinical populations (Kaufman et al. 1997; Gokler et al. 2004).

### 2.3 Biochemical Measures

Venous blood samples were collected between 8:00 and 10:00 a.m. following an overnight fast of at least 10–12 h to minimize the effects of circadian variation and recent food intake on hormone levels. All participants were instructed to refrain from vigorous physical activity and caffeine consumption prior to sampling.

Blood samples were obtained using standard venipuncture techniques and collected into appropriate serum tubes. Following collection, samples were allowed to clot at room temperature and were subsequently centrifuged at 3000 rpm for 10 min to separate serum. The resulting serum aliquots were immediately transferred into polypropylene tubes and stored at −80°C until analysis to preserve biochemical stability.

Fasting serum levels of total ghrelin and NPY were quantified using commercially available enzyme‐linked immunosorbent assay (ELISA) kits, in accordance with the manufacturers' protocols. All assays were performed in duplicate to ensure measurement reliability. Calibration curves were generated for each assay using standard solutions provided in the kits, and concentrations were calculated based on these standard curves.

To ensure analytical precision, both intra‐assay and inter‐assay coefficients of variation (CVs) were maintained below 10% for all measurements. Laboratory personnel conducting the assays were blinded to participants' group status to reduce potential bias.

### 2.4 MRI Acquisition and Cortical Thickness Analysis

Structural brain images were acquired using a 3.0 Tesla MRI scanner equipped with an 8‐channel head coil. High‐resolution T1‐weighted images were collected using a 3D magnetization‐prepared rapid acquisition gradient echo (MPRAGE) sequence with the following parameters: repetition time (TR) = 2300 ms, echo time (TE) = 2.98 ms, inversion time = 900 ms, flip angle = 9°, field of view = 256 × 256 mm, slice thickness = 1 mm, voxel size = 1 × 1 × 1 mm.

Cortical reconstruction and volumetric segmentation were performed with FreeSurfer software (version 6.0). The automated preprocessing pipeline included motion correction, intensity normalization, removal of non‐brain tissue, Talairach transformation, segmentation of subcortical white matter and gray matter structures, tessellation of the gray/white matter boundary, and surface deformation for cortical thickness estimation. Cortical thickness values were extracted for each region of interest (ROI) based on the Desikan–Killiany atlas. All FreeSurfer outputs underwent visual quality control inspection to verify the accuracy of cortical reconstruction and regional parcellation. Scans showing substantial motion artifacts or segmentation inaccuracies were excluded prior to statistical analysis. Regions of interest included the bilateral insula, anterior cingulate cortex, posterior cingulate cortex (PCC), middle orbital cortex, and lateral orbital cortex.

### 2.5 Statistical Analysis

Group differences in demographic, hormonal, psychometric, and cortical thickness measures were examined using analysis of variance (ANOVA) or chi‐square tests, as appropriate. When significant omnibus effects were identified, Bonferroni‐corrected pairwise post hoc comparisons were performed to examine between‐group differences. Multivariate analysis of covariance (MANCOVA) was performed to assess group differences in cortical thickness, controlling for age, sex, and BMI. Post hoc pairwise comparisons following significant MANCOVA findings were also adjusted using Bonferroni correction. Associations between hormone levels, cortical thickness, and psychometric measures were analyzed using Pearson or Spearman correlation coefficients depending on distribution. Variables demonstrating non‐normal distribution were log‐transformed prior to analysis; this procedure was applied to right posterior cingulate cortical thickness values to reduce skewness and improve distributional normality. To control for multiple testing across the 10 cortical regions of interest, *p* values obtained from the ROI analyses were additionally adjusted using the Benjamini–Hochberg false discovery rate (FDR) procedure. Statistical significance following correction was defined as *q* < 0.05.

Exploratory multiple linear regression analyses were conducted across the pooled sample to examine whether BMI and cortical thickness measures in regions implicated in interoception and self‐referential processing predicted NPY levels. Because both NPY levels and cortical thickness measures showed group‐related variation, diagnostic group membership (BED, obesity without BED, healthy controls) was included in the regression model to account for potential between‐group effects. Given the modest sample size and exploratory nature of the analyses, the number of predictors included in the model was intentionally limited to reduce the risk of overfitting. All predictors were entered simultaneously into the model. Multicollinearity was assessed using variance inflation factors (VIF), and all values were below 5, indicating no problematic multicollinearity.

Statistical significance was set at *p* < 0.05 (two‐tailed). All analyses were performed with SPSS version 25.0 (IBM Corp., Armonk, NY, USA).

## 3 Results

### 3.1 Sociodemographic and Clinical Characteristics

Sociodemographic and clinical characteristics of the study groups are summarized in Table 1. The three groups (BED, obesity without BED, and healthy controls) did not differ significantly in terms of age or sex distribution (*p* > 0.05). As expected, BMI was significantly higher in both groups with obesity compared to healthy controls (*p* < 0.001), with no difference between BED and non‐BED adolescents with obesity.

**TABLE 1:** Participant sociodemographic and clinical characteristics.

Variable	BED (*n* = 24)	OB (*n* = 24)	HC (*n* = 22)	*F*/*X* ^2^	*p*	Post hoc
Age, *M* ± SD	15.0 ± 1.8	14.5 ± 1.7	14.5 ± 1.5	0.6	0.537	ns
Sex, female *n* (%)	16 (66.7)	16 (66.7)	15 (68.2)	0.0	0.992	ns
BMI, *M* ± SD	33.6 ± 2.9	34.0 ± 4.1	20.0 ± 2.8	129.3	**< 0.001**	BED=OB>HC
CDI, *M* ± SD	13.4 ± 5.7	11.1 ± 3.9	4.1 ± 2.5	28.9	**< 0.001**	BED=OB>HC
EAT‐40 total Score, *M* ± SD	55.8 ± 22.2	52.6 ± 16.9	17.9 ± 14.7	29.9	**< 0.001**	BED=OB>HC
EDE‐Q restraint eating, *M* ± SD	10.5 ± 6.8	10.6 ± 5.7	3.3 ± 3.6	13.0	**< 0.001**	BED=OB>HC
EDE‐Q eating concern, *M* ± SD	6.7 ± 5.4	7.4 ± 5.6	3.0 ± 3.2	5.3	**0.007**	BED=OB>HC
EDE‐Q weight concern, *M* ± SD	15.2 ± 5.5	14.1 ± 5.0	4.5 ± 4.3	32.7	**< 0.001**	BED=OB>HC
EDE‐Q shape concern, *M* ± SD	23.9 ± 12.0	20.2 ± 9.0	7.1 ± 6.6	19.3	**< 0.001**	BED=OB>HC
EDE‐Q total score (log), *M* ± SD	1.3 ± 0.2	1.3 ± 0.2	1.0 ± 0.1	13.7	**< 0.001**	BED=OB>HC
STAI‐State, *M* ± SD	44.5 ± 5.9	45.0 ± 4.8	41.2 ± 5.2	3.4	**0.039**	ns^a^
STAI‐Trait, *M* ± SD	48.6 ± 5.7	50.2 ± 5.1	45.3 ± 4.2	5.6	**0.006**	BED=OB, BED = HC, OB>HC

Abbreviations: BED, binge eating disorder; BMI, body mass index; CDI, children depression inventory; EAT‐40, eating attitudes test; EDE‐Q, Eating Disorder Examination Questionnaire; HCs, healthy controls; ns, non significant; OB, obesity group; SD, standard deviation.

^a^ The initial significance did not persist in post hoc comparisons.

On psychological measures, both BED and adolescents with obesity without BED scored significantly higher than healthy controls on depressive symptoms (CDI), disordered eating attitudes (EAT‐40), and all Eating Disorder Examination Questionnaire (EDE‐Q) subscales, including restraint eating, eating concern, weight concern, and shape concern (all *p* < 0.01). Post hoc analyses revealed no differences between the BED and groups with obesity in these measures, with both groups consistently showing greater psychopathology compared to healthy peers.

Regarding anxiety, state anxiety scores (STAI‐S) were initially higher in the groups with obesity compared to healthy controls (*p* = 0.039), but this difference did not remain significant after post hoc testing. Trait anxiety scores (STAI‐T) differed significantly between groups (*p* = 0.006), with adolescents with obesity showing higher levels than healthy controls, whereas BED adolescents did not significantly differ from either group.

### 3.2 Appetite‐Related Hormones

No group differences were observed in fasting total ghrelin concentrations (*p* = 0.623). However, NPY levels differed significantly across groups (*F* = 18.0, *p* < 0.001). Both BED and adolescents with obesity without BED exhibited elevated NPY levels compared to healthy controls (Figure 1A), with no significant difference between the two groups with obesity. Group differences in appetite‐related hormone levels and cortical thickness measures are presented in Table 2.

**FIGURE 1:**
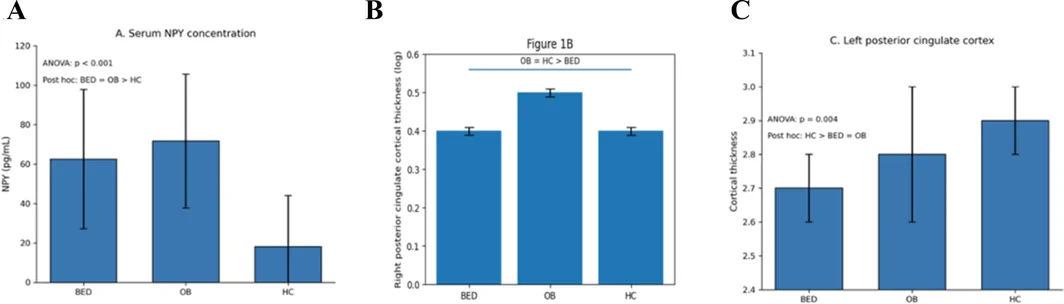
Group differences in appetite‐related hormone levels and cortical thickness measures. (A) Serum neuropeptide Y (NPY) concentrations across groups. (B) Right posterior cingulate cortical thickness across groups. (C) Left posterior cingulate cortical thickness across groups. Error bars represent standard deviations. BED: Adolescents with binge eating disorder and obesity; OB: Adolescents with obesity without BED; HC: Healthy controls.

**TABLE 2:** Group differences in appetite‐related hormone levels and cortical thickness.

Variable	BED (*n* = 24)	OB (*n* = 24)	HC (*n* = 22)	*F*	*p*	Post hoc
Serum ghrelin concentration (pg/mL)	65.3 ± 23.6	64.1 ± 32.4	72.0 ± 31.2	0.5	0.623	ns
Serum NPY concentration (pg/mL)	62.5 ± 35.3	71.7 ± 33.9	18.1 ± 25.9	18.0	**< 0.001**	BED=OB>HC
The R part of insula	6.9 ± 0.6	7.2 ± 0.8	7.2 ± 0.6	1.3	0.285	ns
The L part of insula	7.2 ± 0.7	7.7 ± 0.9	7.5 ± 0.7	2.2	0.119	ns
The R part of caudal anterior cingulate	2.7 ± 0.3	2.7 ± 0.3	2.7 ± 0.3	0.5	0.615	ns
The L part of anterior cingulate	2.7 ± 0.3	2.7 ± 0.3	2.7 ± 0.3	0.6	0.571	ns
The R part of posterior cingulate (log)	0.4 ± 0.0	0.5 ± 0.0	0.4 ± 0.0	6.6	**0.002**	OB=HC>BED
The L part of posterior cingulate	2.7 ± 0.1	2.8 ± 0.2	2.9 ± 0.1	6.0	**0.004**	HC>BED=OB
The R part of middle orbital	6.6 ± 0.8	6.5 ± 0.9	6.5 ± 0.5	0.1	0.943	ns
The L part of middle orbital	6.4 ± 0.8	6.4 ± 0.8	6.3 ± 0.6	0.3	0.753	ns
The R part of lateral orbital	3.7 ± 0.4	3.7 ± 0.6	3.7 ± 0.5	0.2	0.850	ns
The L part of lateral orbital	4.2 ± 0.5	4.4 ± 0.7	4.2 ± 0.5	1.1	0.353	ns

Abbreviations: BED, binge eating disorder; HCs, healthy controls; L, left; ns, non significant; R, right, SD, standard deviation.

### 3.3 Cortical Thickness

Group differences were observed in several brain regions. Adolescents with BED showed reduced cortical thickness in the right PCC compared to both adolescents with obesity without BED and healthy controls (*F* = 6.6, *p* = 0.002; Figure 1B). In addition, left posterior cingulate cortical thickness was reduced in both groups with obesity compared to healthy controls (*F* = 6.0, *p* = 0.004; Figure 1C). The significant group differences observed in the right PCC (*p* = 0.002) and left PCC (*p* = 0.004) remained significant after Benjamini–Hochberg FDR correction across all 10 cortical regions of interest (both *q* < 0.05). No significant group differences were found in cortical thickness of the insula, anterior cingulate cortex, middle orbital cortex, or lateral orbital cortex (all *p* > 0.05).

### 3.4 Exploratory Regression Analysis

Results of the multiple linear regression analysis are presented in Table 3. The regression model remained significant after adjustment for diagnostic group membership (*F*(7,62) = 5.87, *p* < 0.001, adjusted *R* ^2^ = 0.31). BMI remained a significant positive predictor of NPY levels (*β* = 0.47, *p* < 0.001), whereas lower left insular cortical thickness remained independently associated with higher NPY levels (*β* = −0.45, *p* = 0.031). Diagnostic group membership was not independently associated with NPY levels after adjustment for BMI and cortical thickness measures. Inclusion of group membership did not materially alter the pattern of findings.

**TABLE 3:** Multiple linear regression model examining the association between cortical thickness measures, BMI, diagnostic group membership, and plasma NPY levels.

Variable	*β* (95% CI)	*p*
Body Mass Index	0.47 (0.25 to 0.68)	**< 0.001**
The R part of posterior cingulate (log)	0.18 (−0.05 to 0.41)	0.118
The L part of posterior cingulate	−0.09 (−0.33 to 0.15)	0.452
The R part of insula	0.32 (−0.10 to 0.74)	0.134
The L part of insula	−0.45 (−0.85 to −0.04)	**0.031**
BED (vs. healthy controls)	0.14 (−0.18 to 0.46)	0.387
Obesity without BED (vs. healthy controls)	0.11 (−0.20 to 0.42)	0.476

*Note:* Diagnostic group membership was entered using dummy coding, with healthy controls as the reference category. *R* ^2^ = 0.38; Adjusted *R* ^2^ = 0.31.

Abbreviations: BED, binge eating disorder; BMI, body mass index; NPY, neuropeptide Y.

## 4 Discussion

The present study examined the interplay between cortical thickness and appetite‐regulating hormones in adolescents with obesity ± BED, providing an integrated neurobiological perspective on disordered eating during a critical developmental period. First, both groups with obesity showed elevated NPY levels compared to healthy controls, independent of BED status. Second, adolescents with BED demonstrated additional structural alterations, particularly reduced cortical thickness in the right PCC, while left PCC thinning was observed across both groups with obesity. Finally, regression analyses revealed that higher BMI and reduced insular cortical thickness were independently associated with elevated NPY levels.

One of the most notable findings is the elevation of NPY in both groups with obesity, suggesting that dysregulation of orexigenic signaling may represent a core neurobiological feature of adolescent obesity rather than a BED‐specific mechanism (Vähätalo et al. 2015). NPY is a potent hypothalamic peptide involved in energy balance and food intake regulation, and its elevation has been consistently linked to increased appetite and weight gain (Assan et al. 2021). The absence of differences between groups with obesity ± BED may indicate that NPY‐related pathways contribute more to general overeating tendencies than to the episodic loss of control that characterizes BED (Huang et al. 2021). The absence of significant ghrelin differences across groups may also reflect the heterogeneous and inconsistent findings reported in prior BED literature (Camacho‐Barcia et al. 2026). This aligns with prior literature suggesting that while neuroendocrine alterations are central in obesity, the differentiation of BED may depend more heavily on higher‐order cognitive and emotional processes (Camacho‐Barcia et al. 2026).

In contrast, structural neuroimaging findings point to more specific alterations associated with BED. Reduced cortical thickness in the right PCC in adolescents with BED is particularly noteworthy, given the role of this region in self‐referential processing, internal mentation, and aspects of reward evaluation (Leech and Sharp 2014). The PCC is a central node of the default mode network (DMN), which has been implicated in maladaptive rumination and altered self‐related cognition in various psychiatric conditions (Menon 2023). In the context of BED, reduced PCC thickness may reflect impairments in the integration of internal states and behavioral regulation, potentially contributing to the subjective loss of control during binge episodes (Duck et al. 2025). The finding that left PCC thinning was present in both groups with obesity further suggests that obesity itself may be associated with broader alterations in DMN‐related structures, while additional right‐sided changes may differentiate BED. The absence of significant differences in EDE‐Q and EAT‐40 scores between adolescents with and without BED suggests that the observed cortical thickness differences should be interpreted cautiously. Although right posterior cingulate alterations were observed specifically in the BED group, these findings may reflect categorical BED diagnosis, loss‐of‐control eating characteristics, or other neurobehavioral features not fully captured by dimensional eating‐disorder psychopathology measures. Therefore, distinctions between BED‐related and obesity‐related neurobiological alterations remain preliminary. Notably, the PCC has not consistently emerged in prior structural neuroimaging studies of adolescent obesity or adult BED (Leech and Sharp 2014). Therefore, these findings should be interpreted cautiously and may reflect developmental, sample‐specific, or methodological characteristics requiring replication in larger longitudinal studies.

Interestingly, no significant group differences were observed in the insula or anterior cingulate cortex, regions frequently implicated in interoception, salience processing, and inhibitory control (Wang et al. 2019). This may indicate that structural alterations in these regions are either subtler in adolescence or more functionally than morphologically expressed. Alternatively, it is possible that functional abnormalities precede detectable structural changes in these regions during early stages of disordered eating.

The regression findings provide further insight into the relationship between neuroendocrine and neurostructural factors. The independent association between higher BMI and elevated NPY levels supports the well‐established link between adiposity and orexigenic signaling (Tang et al. 2020). More importantly, the negative association between insular cortical thickness and NPY suggests a potential interaction between peripheral metabolic signals and central interoceptive processing (Wang et al. 2019). The insula plays a key role in representing internal bodily states, including hunger and satiety, and reduced cortical thickness in this region may impair the accurate interpretation of physiological signals, thereby contributing to dysregulated eating behavior (Livneh et al. 2017). This finding suggests a potential association between hormonal regulation and cortical structure reinforcing each other. Importantly, the association between NPY levels and left insular cortical thickness remained significant after adjustment for diagnostic group membership, suggesting that the observed relationship was not solely attributable to between‐group differences. Nevertheless, these findings should be interpreted cautiously given the exploratory and cross‐sectional nature of the analyses.

Interestingly, contrary to several prior studies, adolescents with BED and obesity did not differ significantly from adolescents with obesity without BED in EDE‐Q or EAT‐40 scores (Turan et al. 2021). This may reflect overlapping eating‐related psychopathology across adolescents with obesity during a developmental period characterized by heightened body‐related concerns and dysregulated eating behaviors. Additionally, the relatively small sample size and the use of strict DSM‐5 BED criteria may have reduced sensitivity to detect more subtle psychopathological differences between groups.

From a developmental perspective, these findings are particularly relevant. Adolescence is characterized by ongoing maturation of neural circuits involved in reward processing, impulse control, and emotional regulation. The coexistence of heightened orexigenic signaling and structural alterations in self‐referential and interoceptive brain regions may create a neurobiological vulnerability that increases the risk of persistent disordered eating patterns (Hagan et al. 2025). This underscores the importance of early identification and intervention in adolescents with obesity and BED.

Several limitations should be considered when interpreting these results. First, the cross‐sectional design precludes conclusions about causality or developmental trajectories. Longitudinal studies are needed to determine whether hormonal alterations precede cortical changes or vice versa. Second, the sample size, although comparable to prior neuroimaging studies, may limit statistical power for detecting subtle regional differences. A diagnostic consideration is that DSM‐5‐based BED criteria requiring objectively large binge‐eating episodes were used, which may limit generalizability to adolescents with subthreshold BED or broader loss‐of‐control eating presentations commonly included in age‐adapted pediatric definitions. Third, only two appetite‐related hormones were assessed; inclusion of additional markers such as leptin or insulin could provide a more comprehensive understanding of metabolic regulation. Another limitation is the absence of a BED group without obesity, which limits the ability to distinguish BED‐specific alterations from neurobiological changes associated with obesity itself. Finally, functional neuroimaging data were not included, which could have complemented the structural findings.

Despite these limitations, the present study has several strengths, including the integration of neuroimaging, biochemical, and psychometric data in a well‐characterized adolescent sample. By simultaneously examining cortical structure and appetite‐related hormones, this study advances current understanding of the neurobiological mechanisms underlying obesity and BED in youth.

In conclusion, our findings suggest that elevated NPY levels may represent a shared feature of adolescent obesity, whereas structural alterations in the right PCC may distinguish adolescents with BED. The observed associations between cortical thickness and hormonal regulation further highlight the complex interaction between brain structure and metabolic processes. These results support a multidimensional model of BED in adolescence and may have implications for the development of targeted interventions addressing both neurobiological and behavioral components of disordered eating.

## Author Contributions

**Serkan Turan:** conceptualization, investigation, funding acquisition, writing – review and editing, methodology, formal analysis, supervision, data curation, writing – original draft. **İbrahim Mert Erbaş:** conceptualization, investigation, funding acquisition, validation, methodology. **Fatma Ceren Sarıoğlu:** conceptualization, investigation, writing – original draft, writing – review and editing, software. **Berrin Çavuşoğlu:** conceptualization, investigation, funding acquisition, validation, visualization, software. **Ezgi Molla:** conceptualization, methodology, software, investigation, validation, funding acquisition, writing – original draft, writing – review and editing. **Ali Rıza Şişman:** conceptualization, methodology, investigation, validation, formal analysis. **Sevay Alsen Güney:** conceptualization, investigation, validation, formal analysis, project administration, writing – review and editing, writing – original draft. **Handan Güleryüz:** conceptualization, investigation, writing – original draft, writing – review and editing, validation. **Ayhan Abacı:** conceptualization, investigation, validation, visualization, formal analysis. **Yeşim Öztürk:** conceptualization, investigation, funding acquisition, visualization, validation, formal analysis. **Aynur Pekcanlar Akay:** conceptualization, investigation, writing – original draft, funding acquisition, validation, visualization, methodology, project administration.

## Lived Experience Involvement Statement

No specific efforts were undertaken to involve persons with lived experience in the study design or execution, or in the preparation of this manuscript.

## Funding

This study was supported by a grant from the Dokuz Eylül Üniversitesi/Dokuz Eylul University Department of Scientific Research Projects (2017.KB.SAG.051).

## Conflicts of Interest

The authors declare no conflicts of interest.

## Data Availability

The data that support the findings of this study are available on request from the corresponding author. The data are not publicly available due to privacy or ethical restrictions.
